# Green Synthesis of Ag Nanoparticles Using Grape Stalk Waste Extract for the Modification of Screen-Printed Electrodes

**DOI:** 10.3390/nano8110946

**Published:** 2018-11-17

**Authors:** Julio Bastos-Arrieta, Antonio Florido, Clara Pérez-Ràfols, Núria Serrano, Núria Fiol, Jordi Poch, Isabel Villaescusa

**Affiliations:** 1 Departament d’Enginyeria Química, Escola d’Enginyeria de Barcelona Est (EEBE), Universitat Politècnica de Catalunya, BarcelonaTEch (UPC), Av. Eduard Maristany 16, 08019 Barcelona, Spain; antonio.florido@upc.edu; 2 Physical Chemistry TU Dresden, Zellescher Weg 19, 01062 Dresden, Germany; 3 Barcelona Research Center for Multiscale Science and Engineering, Av. Eduard Maristany 16, 08019 Barcelona, Spain; 4 Departament d’Enginyeria Química i Química Analítica, Facultat de Química, Universitat de Barcelona, c/Martí i Franquès 1-11, 08028 Barcelona, Spain; claraperezrafols@ub.edu (C.P.-R.); nuria.serrano@ub.edu (N.S.); 5 Departament d’Enginyeria Química, Escola Politècnica Superior, Universitat de Girona, c/Ma Aurèlia Capmany, 61, 17071 Girona, Spain; isabel.villaescusa@udg.edu; 6 Departament d’Informàtica Aplicada i Matemàtiques, Escola Politècnica Superior, Universitat de Girona, c/Ma Aurèlia Capmany, 61, 17071 Girona, Spain; jordi.poch@udg.edu

**Keywords:** silver nanoparticles, screen-printed electrodes, grape stalk, green synthesis, voltammetry, metal analysis

## Abstract

The chemical synthesis of silver nanoparticles (Ag-NPs) by using an environmentally friendly methodology for their preparation is presented. Thus, considering that plants possess components that can act as reducing agents and stabilizers in nanoparticles’ production, the synthesis of Ag-NPs by using an extract aqueous solution of grape stalk waste as a reducing and capping agent is studied. First, the total polyphenols and reducing sugars contained in the produced extracts at different conditions are characterized. After that, Ag-NPs are synthesized regarding the interaction of Ag ions (from silver nitrate) and the grape stalk extract. The effect of temperature, contact time, extract/metal solution volume ratio and pH solution in the synthesis of metal nanoparticles are also studied. Different sets of nanoparticle samples are characterized by means of Electron Microscopy coupled with Energy Dispersive X-Ray for qualitative chemical identification. Ag-NPs with an average diameter of 27.7 ± 0.6 nm are selected to proof their suitability for sensing purposes. Finally, screen-printed electrodes modified with Ag-NPs are tested for the simultaneous stripping voltammetric determination of Pb(II) and Cd(II). Results indicate good reproducibility, sensitivity and limits of detection around 2.7 µg L^−1^ for both metal ions.

## 1. Introduction

Several recent publications have reviewed the greener routes to metal nanoparticles synthesis mediated by microorganisms [1], algae and waste material [2], and plant material extract [3,4]. The different routes to synthesize silver nanoparticles (Ag-NPs) have also been reviewed recently. All these routes comprise three steps: silver reduction by bioreducing agents, agglomeration and stabilization of NPs [5]. Some authors underline the advantages of using plant derivatives extracts over microorganisms. Plant derivatives extracts have no need for cells culture maintenance nor sterile conditions [6,7]. Moreover, plant extracts provide natural capping agents for the stabilization of nanoparticles, reducing the nanoparticles synthesis process to a single step [8]. All these advantageous features of plant extracts result in less reagent consumption and, consequently, in a reduction of the cost that facilitates the development of large scale production of nanoparticles in an environmentally friendly approach. The synthesis of silver nanoparticles mediated by plant extracts, recently reviewed by Ahmed [8] and Kuppusamy [9], reveals that reduction and stabilization of the nanoparticles is performed by a combination of different biomolecules such as proteins, amino acids, enzymes, polysaccharides, alkaloids, tannins, phenolic acid, terpenoids and flavonoids. The content of these biomolecules acting as reducing and capping agents is known to be dependent on the source of the extract, providing different characteristics (i.e size, shape, stability) to the synthesized nanoparticles. In spite of the importance of the extract composition on the reduction process, few researchers reporting nanoparticles biosynthesis provide information about the main reducing components of the extract. 

Grape stalks (GS) are the lignocellulosic skeleton of grape and are the main by-product after grape harvesting and wine production. The extract of some grape sub-products such as leaves, stems, seeds and dried fruit, have been used for the production of bimetallic Fe/Pd [10], Fe_3_O_4_-Ag [11] and silver [12] nanoparticles, respectively. Three different grape wastes (seed, skin and stalk) were used by Krishnaswamy et al. [13], for the production of gold nanoparticles. A chemical characterization of grape stalk carried out recently [14] revealed that the main components of this by-product are polar compounds soluble in hot water with a high content in tannins and other polyphenolic compounds. These results suggested that GS extract would also contain polyphenolic compounds that could act as reducing and stabilizing agents in the nanoparticles formation. 

Nanoparticles (NPs) have been widely applied in different research fields due to their surface-volume ratio that provides them with unique and improved properties in comparison to the analogous bulk material. One interesting feature of NPs (e.g., Ag-NPs) is the simplicity of their application in the modification of screen-printed electrodes (SPEs). Nowadays, this results in a great platform for environmental portable electroanalysis due to their commercial availability, relatively low cost and reproducibility. Nanoparticles enhance the electron transfer among redox centers between the analyte and the electrode, decreasing the over potentials of several analytically important electrochemical reactions [15,16]. Although the use of NPs has already been successfully applied to the development of new electrochemical sensors for the determination of heavy metal ions [16,17,18], to the best of our knowledge, no previous research about the implementation of green-synthetized NPs has been published.

Here we report the synthesis of silver nanoparticles (Ag-NPs) mediated by the use of aqueous extract of grape stalk waste, which results in a non-reagent procedure and in the valorization of an agro-food waste. The evaluation of different factors that affect the synthesis of these Ag-NPs is also performed. Moreover, and for the first time, the feasibility of these green synthesized Ag-NPs for electrochemical assays is presented. For this purpose, screen-printed carbon nanofibers electrodes (SPCNFE), which have been demonstrated to be a suitable support for the modification of sensors [19], are modified with Ag-NPs by using the drop-casting approach. The resulting modified sensor is applied to the simultaneous determination of Cd(II) and Pb(II) ions by differential pulse anodic stripping voltammetry (DPASV) in aqueous solution as a model electrochemical metal ion system.

## 2. Materials and Methods

### 2.1. Reagents and Materials

Grape stalk waste from wine production was kindly supplied by a winemaking cooperative (Empordà-Costa Brava, Spain). The waste was rinsed abundantly with water, dried and cut to separate the pedicels from the stalk branches. The pedicels were then ground and sieved to get the desired particle size (1–1.6 mm). Chemical reagents were acquired from Sigma Aldrich (Munich, Germany) as AgNO_3_ for the preparation of Ag nanoparticles, Pb(NO_3_)_2_·4H_2_O and Cd(NO_3_)_2_·4H_2_O, for the preparation of 10^−2^ mol L^−1^ Cd(II) and Pb(II) stock solutions respectively, and standardized complexometrically [20]. 0.1 mol L^−1^ acetate buffer solution (pH 4.5) was used for pH control. Ultrapure water (Milli-Q plus 185 system, Millipore, Burlington, MA, USA) was used in all experiments.

### 2.2. Preparation of Grape Stalk Extract

The extract was obtained by placing grape stalk waste in a 200 mL round bottom flask and adding 100 mL of Milli-Q water. A reflux condenser was placed at the top of the flask. The device was placed in a heating mantle. Extracts were obtained at different temperatures (60–100 °C) and contact times (15–120 min). After this, the extract was separated from the solid by filtration using a cellulose paper and then centrifuged for 30 min at 5000 rpm (Universal 320, Selecta). Extract samples were analyzed in order to determine their content in total polyphenols and reducing sugars. Prior to the analysis, the loss of volume of the filtrate due to possible water evaporation was compensated by adding water to a total volume of 100 mL.

#### Determination of Total Phenolic and Reducing Sugars

In total, 34 extracts were prepared at different experimental conditions (temperature and contact time). The total polyphenol content of the filtered extracts was analyzed by the Folin-Ciocalteu method and the reducing sugars content by reaction of the sample with Fehling’s solution followed by iodimetric titration of the unreduced copper remaining in the solution. A statistical analysis of experimental data (total polyphenols and reducing sugars) was performed to determine which of the studied parameters (temperature and extraction time) had a significant influence on the concentration of total polyphenols and reducing sugars of the obtained extracts. This statistical analysis was performed using SPSS software program for Windows with a significance level of 0.05 (confidence interval 95%). 

### 2.3. Synthesis of Silver Nanoparticles Using Grape Stalk as Reducing Agent

Synthesis of silver nanoparticles was carried out by putting into contact a certain volume of extract with a certain volume of 0.01 mol L^−1^ silver nitrate solution in sample tubes. The tubes were immersed in a thermostatic water bath (Digiterm 3000542, Selecta, Abrera, Spain) adjusting to the studied temperature (60 °C, 70 °C, 80 °C and 100 °C). The color change from pale yellow brown to reddish brown indicated the reduction of silver nitrate to metallic silver. This color change has been taken as indicative of Ag-NPs synthesis by almost all the researchers [5,21]. Once the samples were cooled down, pH of the solution was measured (pH meter Basic 20, Crison Instruments, Alella, Barcelona, Spain). Then, the samples were first filtered through Whatman No. 1 filter paper and then centrifuged at 5000 rpm for 30 min. The pellet was kept in the fridge for further analysis and the maximum absorption of the supernatant was scanned at the wavelength between 200–800 nm. 

### 2.4. Characterization of Ag Nanoparticles

#### 2.4.1. UV/Vis Spectroscopy

The UV/Vis spectra of the centrifugation supernatants diluted 1:10 were recorded using an UVmini-1240 spectrophotometer (Shimadzu, Kyoto, Japan). Different spectra were obtained showing the Surface Plasmon Resonance (SPR) of the prepared Ag-NPs. 

#### 2.4.2. Electron Microscopy Characterization

Spectrometer (EDX, Oxford Instrument, Oxon, United Kingdom), Zeiss^®^ Gemini SEM/Scanning Transmission Electron (STEM) with Focus Ion Beam (FIB-SEM, Zeiss, Berlin, Germany), and High Resolution Transmission Electron Microscopy JEM 2011 with an acceleration voltage of 200 kV (HR-TEM, JEOL, Nieuw-Vennep, The Netherlands) coupled to an EDX (Oxford Instrument, Oxon, United Kingdom ) were used. Approximately 1 mL of sample was filtered and then dispersed in 5 mL of acetone as organic solvent and then placed in an ultrasound bath for 1 h. Finally, 50 μL of the solution were placed on a grid and dried before TEM analysis. For SEM analysis, 50 μL of NPs dispersion were drop-casted on a silicon wafer and then attached to a SEM sample stub with a small piece of carbon conductive tape. For STEM images, TEM grid was placed on a special sample stub holder. 

The size distribution of the prepared NPs was obtained by direct observation of the TEM images and the construction of the corresponding size distribution histograms. These measurements were performed using the Image J Software, and the obtained histograms were adjusted to a three-parameter Gaussian curve (Equation (1)) where *x*_0_ is the mean diameter (to which most nanoparticles correspond), *b* is the standard deviation and *a* is a statistical parameter related to this fitting. Histograms were calculated using the Microsoft Excel 2010, and the equation was adjusted with SigmaPlot 11.0.

(1) $$\;y=ae^{\left[-0.5{\left(\frac{x_{0}-x}{b}\right)}^{2}\right]}\;$$

#### 2.4.3. Fourier Transformed Infrared (FTIR) Spectroscopy

To elucidate the functional groups, present in the grape stalk extract and the molecules surrounding the Ag nanoparticles, FTIR (Bruker, Ettlingen, Germany) spectra in the range of 4000–400 cm^−1^ were performed in a Platinum ATR spectrometer (Bruker, Ettlingen, Germany). Both raw extract and three times washed and centrifuged Ag-NPs (to ensure the removal of unbound molecules) were freeze dried in a lyophilizer Unitop HL (Virtis, SP Scientific, Ipswich, UK).

#### 2.4.4. Electrochemical Characterization of Modified SPCNFE

Differential pulse anodic stripping voltammetric (DPASV) measurements were performed in an Autolab System PGSTAT12 (EcoChemie, Utrecht, The Netherlands), in a multichannel configuration, attached to a Metrohm 663 VA Stand (Metrohm, Herisau, Switzerland) and a personal computer with GPES Multichannel 4.7 software package (EcoChemie, Utrecht, The Netherlands).

The auxiliary and the reference electrode (to which all potentials are referred) were Pt wire and Ag|AgCl|KCl (3 mol L^−1^), respectively, both purchased from Metrohm (Herisau, Switzerland). The working electrode used was a screen-printed carbon nanofiber electrode (SPCNFE) modified with silver nanoparticles (Ag-NPs-SPCNFE) and connected to the Autolab Systems by means of a flexible cable (ref. CAC, Dropsens, Oviedo, Spain). SPCNFE was a disk electrode of 4 mm diameter provided by Dropsens (ref. 110CNF, Dropsens, Oviedo, Spain). 

Stripping voltammetric measurements using Ag-NPs-SPCNFE for the determination of Pb(II) and Cd(II) ions were carried out at a deposition potential (E_d_) of −1.40 V applied with stirring during a deposition time (t_d_) of 120 s and followed by a rest period (t_r_) of 5 s. Measurements were performed by scanning the potential from −1.40 to −0.30 V, and by using pulse times of 50 ms, step potentials of 5 mV and pulse amplitudes of 50 mV. All experiments were carried out without any oxygen removal and at room temperature (20 °C). 

Ag-NPs-SPCNFE working electrodes were prepared by drop-casting 40 µL of Ag-NPs solution onto the electrode surface and drying it at 50 °C for 30 min. The Ag-NPs solutions were supernatants resulting from different washings of the pellets obtained from Ag-NPs carried out at different pH values (pH 4 and 6). The corresponding pellets were suspended in 10 mL of deionized water under agitation. Then the suspension was centrifuged. The resulting supernatant (W1) was separated from the washed pellet. This washing operation was repeated three more times. The aim of these washings was to remove non-reacted ions and molecules from the silver nanoparticles suspension that could interfere on the sensor response. The obtained supernatants (W1, W2, W3 and W4, increasing regarding the number of times the pellets were washed) obtained from NPs synthesis, were drop-casted on SPCNFE to obtain 16 different modified sensors.

## 3. Results and Discussion

### 3.1. Grape Stalk Extracts

**Figure 1:**
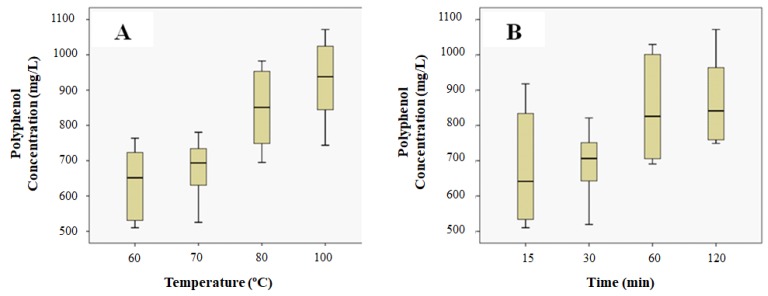
BP for the concentration of polyphenols at different temperatures (**A**) and contact times (**B**).

To show the effect of temperature and contact time on grape stalk extraction, box plots (BP) were used (Figure 1). The line across the box represents the median value, whereas the top and the bottom box show the location of the first and third quartiles (Q1 and Q3). The whiskers are the lines that extend from the top and bottom of the box. The box itself contains the middle 50% of the data. If the median is not equidistant from the top and the bottom of the box, then the data are skewed. BP corresponding to total polyphenol concentration values obtained at different temperatures and at different contact times are presented in Figure 1A,B, respectively.

From the results of the BP, a one-way ANOVA test was performed to analyze the differences among group means and their variation, specifically for the concentration of polyphenols and reducing sugars at different temperatures and different extraction times. The results of the ANOVA test (results not presented) showed *p* < 0.05 for the concentration of polyphenols at different temperatures and contact times and also for the concentration of reducing sugars at different contact times. However, *p* > 0.05 for the concentration of reducing sugars at different temperatures was obtained. Bonferroni post hoc test was carried out to seek out the variable responsible for significant differences between the groups of data. Results indicated that temperature is a significant variable for polyphenols concentration: *p*-values for the comparison between the temperatures 60 and 70 °C and 80 and 100 °C are above 0.05. Therefore, the temperature significance lies between these two groups (70–80 °C). In the case of the variable extraction time, *p*-values were also all above 0.05 for the polyphenols and almost all below this value for the reducing sugars. These results indicate that contact time does not play a significant role in the polyphenols concentration (around 0.5 g L^−1^) but this variable does have a significant influence in the concentration of reducing sugars (from 0.4 to 0.9 g L^−1^).

### 3.2. Silver Nanoparticles Synthesis

#### 3.2.1. FTIR Characterization of Grape Stalk

**Figure 2:**
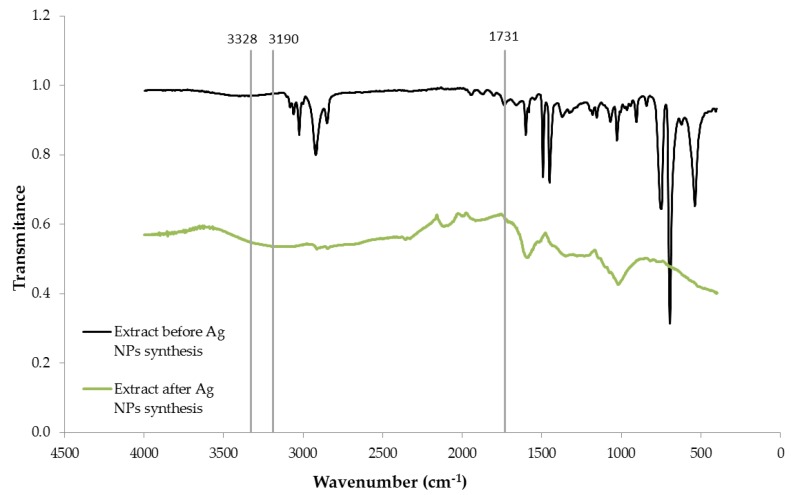
FTIR spectrum of dried grape stalks extract before and after the synthesis of Ag-NPs.

The FTIR spectra of grape stalk (GS) extract before and after the synthesis of Ag-NPs are plotted in Figure 2. The two curves present a high variation in the intensity of bands in most of the regions of the spectrum. Dried extract spectrum displays a number of adsorption peaks indicating the complex nature of this material. The broad peak in the extract spectrum at around 3328 cm^−1^ is indicative of O–H stretching vibrations. The two peaks at 2950 and 2887 cm^−1^ correspond to the asymmetric and symmetric vibration, respectively, of C-H in the olephinic chains and the peak at 1731 cm^−1^ is attributed to the carbonyl C=O in ester groups [14]. The peaks at 1602 and 1524 cm^−1^ are due to vibration of benzene and the peaks at 1132, 1106, 1067 and 1048 can be due to C–O stretching of alcohols and phenols [22].

Dried extract spectrum after the synthesis of Ag-NPs shows a broad band in the region of OH-stretching vibrations around 3190 cm^−1^. The shift from 3328 to 3190 cm^−1^ must be attributed to the formation of Ag-NPs. The observed broadening of the band is characteristic of hydrogen bonding (intra and inter molecular bonds). Free hydroxyl groups might interact with silver to stabilize Ag-NPs. The functional groups p-OH, m-OH and COOH of phenolic groups have been reported to act as hydrogen donors/acceptors [23,24]. A cooperative association between phenolic compounds and other components of the extract may contribute to the stabilization of the nanoparticles [25]. The band at 1731 cm^−1^ attributed to C=O stretching mode has disappeared, indicating that this group is involved in silver reduction and/or NPs stabilization. The bands corresponding to alcohol and phenol stretching shifted to lower wavenumbers and showed a very weak intensity.

#### 3.2.2. Study of Factors Affecting Ag-NPs Synthesis

Some physical and chemical parameters such as pH, contact time between extract and metal solution, temperature, extract/metal solution (*v*/*v*) ratio and metal salt concentration largely affect the synthesis process and the characteristics (size, shape and morphology) of the synthetized NPs [5]. Therefore, the influence of these parameters on Ag-NPs synthesis mediated by GS extract was investigated. Visual observation of color, changes in the pH and spectra of UV-Vis were useful tools to monitoring Ag-NPs formation.

##### Effect of pH

The influence of pH on Ag-NPs was investigated adjusting the pH of the extract at different pH within 2.0–9.0, meanwhile the pH of 0.01 mol L^−1^ AgNO_3_ solution was kept unaltered. In these experiments, the volume of extract and Ag solutions was 4 and 6 mL, respectively, the temperature 80 °C and the contact time 2 h.

The nanoparticles suspension obtained from rinsing the pellet up to three times with Milli-Q water, showed maximum absorbance at around 450 nm, indicating the presence of Ag nanoparticles. It has been reported that silver colloids exhibit maximum absorbance within the range 400–500 nm due to Surface Plasmon Resonance (SPR) [26,27,28].

**Figure 3:**
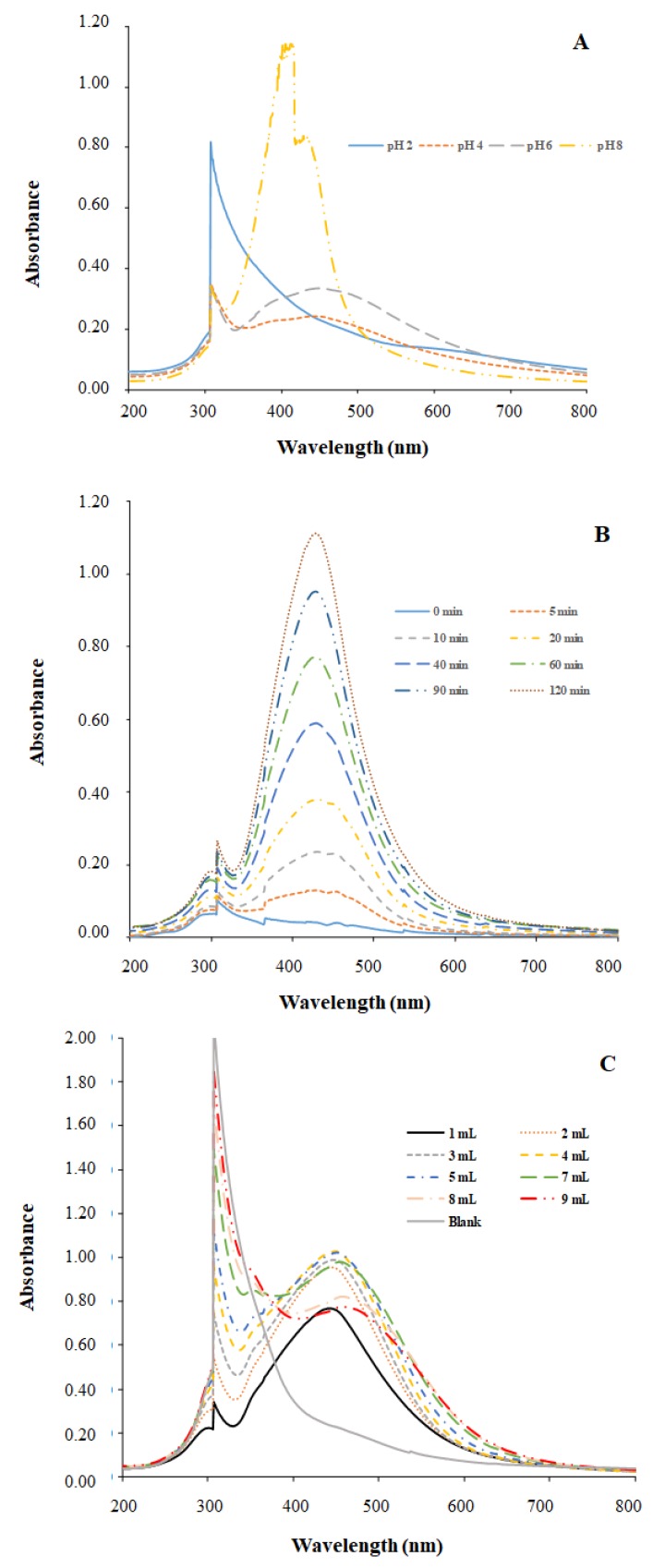
UV/Vis Spectra showing the influence of different parameters in the synthesis of Ag-NPs with the GS extract: (**A**) pH, (**B**) contact time and (**C**) extract addition.

As can be seen in Figure 3A, a peak at 307 nm was observed. This peak must be attributed to the organic matter of the GS extract. This peak presents the higher absorbance value in the solution at pH 2, when no other peak was clearly observed. In the case of pH 4 and 6, a peak around 450 nm was observed. This peak can be attributed to the SPR of silver nanoparticles as mentioned in Section 2.4.1. It must be remarked that the peak absorbance from solution at pH 6 was higher than at pH 4. As peak intensity could be related to Ag-NPs concentration, the increase on peak intensity of solution at pH 6 compared to pH 4 could indicate higher nanoparticles presence in solution. Two peaks were observed in the UV-Vis plot of pH 8 solution and both peaks are in the Ag-NP SPR range. The wavelength of the band shifted to 470 nm when the reaction pH was 4 and 6. The shift towards larger wavelengths (red shift) indicates an increase in the mean diameter of Ag-NPs [29,30].

The high band observed at 440 nm seems to indicate a great number of Ag-NPs. Additionally, the shift towards lower wavelengths indicates a decrease in the mean diameter of Ag NPs [25,26]. Thus, SPR seems to indicate a great number of smaller diameter Ag-NPs when synthesis takes place at pH 8. Nevertheless, the additional peak observed at 415 nm indicates the presence of other compounds in solution that could affect Ag-NPs quality.

##### Effect of Contact Time

In order to ascertain the time required for the completion of the reduction reaction, several tubes containing 4 mL of extract and 6 mL of 0.01 mol L^−1^ AgNO_3_ were heated at 80 °C. The tubes were removed from the thermostatic bath at different times ranging from 5 min to 8 h.

The UV-Vis spectra of Ag-NPs solution obtained at different reaction times are depicted in Figure 3B. In the experimental conditions used (4 mL extract, 6 mL 0.01 M AgNO_3_, 80 °C, pH 4) the Ag-NPs SPR band is noticeable after 5 min of reaction. Up to this reaction time the absorbance intensity increased with time, suggesting a great number of nanoparticles in the solution. 

The pH of the reaction mixture was measured before and after the reduction reaction. The initial pH (4.01) increased in the first 5 min to 4.15 and then progressively diminished with time and reached the pH value of 3.02 after 8 h. The pH change might be related to the involvement of some compounds in the reduction or capping processes leading to Ag-NPs formation.

##### Effect of Extract/Silver Solution (*v*/*v*) Ratio

Different volumes of extract and silver solution ratios were investigated in order to determine the effect of silver addition and the effect of extract addition, respectively. The first one consisted in fixing the volume of extract (4 mL) and adding different volumes (1–6 mL) of 0.01 mol L^−1^ AgNO_3_. In the second one, the volume of silver solution was fixed (1 mL) and the volume of the extract was varied (1–9 mL). For both sets of experiments, the total volume in the tubes was adjusted to 10 mL by adding Milli-Q water. Heating temperature of 80 °C and contact time of 2 h were kept as before.

The plot of the obtained spectra at increasing volumes of extract is presented in Figure 3C. The reacted solutions exhibited an intense dark reddish-brown color with independence of the volume of extract added to 1 mL 0.01 mol L^−1^ AgNO_3_. The increasing sharp peak at 307 nm refers to light absorption of extract components at this wavelength. As expected, absorbance intensity at this wavelength increases with the addition of more volume of extract. Maximum absorbance intensity of the SPR band increased with the addition of 1 to 5 mL of extract. Higher volumes of extract led to the opposite trend as absorbance values decreased with the extra extract addition. The decrease of absorbance could be due to the deficiency of silver ions with respect to the amount of biomolecules that could provoke the agglomeration of smaller particles to form larger Ag-NPs that would absorb light to a lower extent [29]. SPR band values shifted from 447.5 to 459.0 nm with the addition of extract volume from 1 to 9 mL, indicating the effect of extract concentration in the mean diameter of Ag-NPs. López-Carrillo et al. [31] reported, that in general, particle size can be controlled by changing the volume of the reducing agent.

#### 3.2.3. Electron Microscopy Characterization

**Figure 4:**
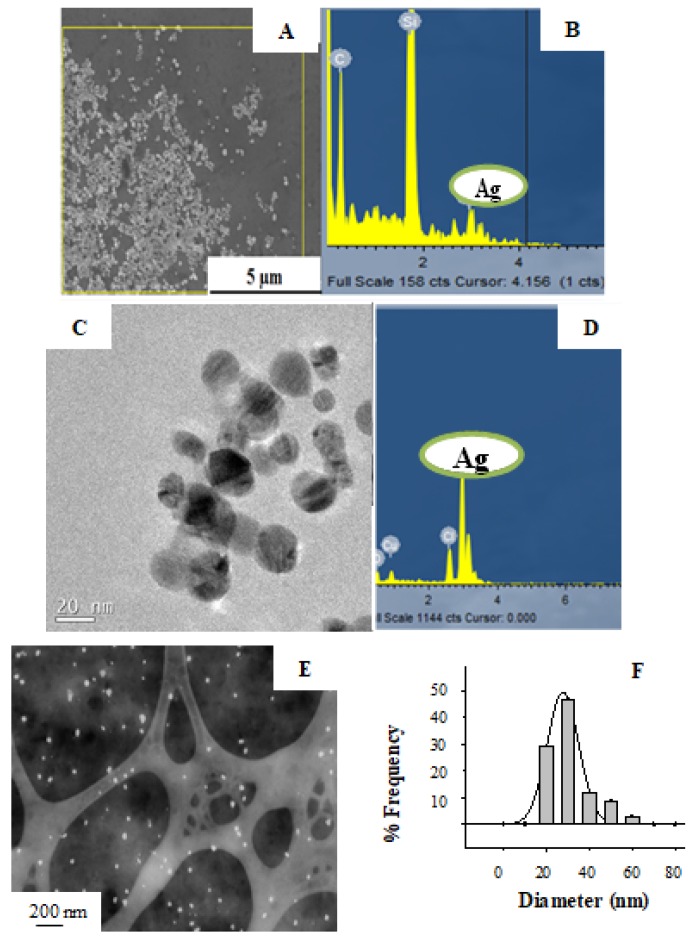
Electron Microscopy micrographs for Ag-NPs synthesized at pH 4 using the GS extract as reducing agent. SEM micrograph (**A**), corresponding EDX spectra (**B**) and TEM micrograph (**C**) with corresponding EDX spectra (**D**) showing the presence of silver. STEM image of Ag-NPs (**E**) with size distribution histogram (**F**).

The suitability of the GS extract in the synthesis of Ag-NPs was proved by direct observation of SEM and TEM images. As an example, Figure 4 shows the electron microscopy characterization of a sample solution of Ag-NPs synthetized at pH 4. Results reveal that Ag-NPs obtained through this methodology presented an average diameter of (27.7 ± 0.6) nm. Moreover, the chemical identity of the Ag-NPs was proved by EDX spectra as it is shown in Figure 4B,D.

The size distribution analysis for the different Ag-NPs synthetized at different pH and measured by TEM images was determined and it is summarized in Table 1. Due to the higher surface-to-volume ratio, these Ag-NPs can present higher electrocatalytical activity. In consequence, they could be suitable for the modification of electrodes, and will be addressed in the following section. 

As can be seen, the smaller particles size of Ag-NPs was obtained when synthesis was carried out at pH 8. This confirms the result of the pH effect on Ag-NPs synthesis analyzed by UV-VIS spectra (see above). Similar nanoparticle diameters were obtained when synthesis was carried out at pH 2 and pH 6. Thus, TEM results ratify the effect of the pH on nanoparticle size. Nevertheless, other aspects related to size stabilization (Ag^+^ ion concentration as NP precursor, concentration of reducing-stabilizing agent, temperature of reaction) seem to have a greater influence than pH on particle size, as no clear trend between pH and size was observed.

**Figure 5:**
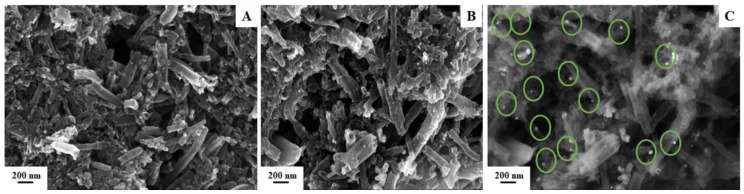
SEM InLens images of raw SPCNFE (**A**) and SPCNFE after the drop casting of Ag-NPs (**B**). SEM Secondary Lens of SPCNFE highlighting the location of the drop-casted Ag-NPs (**C**).

Figure 5 presents SEM images of the non-modified (Figure 5A) and Ag-NPs-modified (Figure 5B,C) SPCNFEs. One can observe that the drop-casting strategy does not alter the morphology of the electrode, but leads to the incorporation of the green synthesized Ag-NPs, distributing all over the surface (Figure 5C). This fact is relevant for the enhancement of the electroanalytical features of the SPCNFE. 

#### 3.2.4. Electrochemical Characterization

The selected E_d_, t_d_ and t_r_ were firstly optimized to ensure the simultaneous determination of Cd(II) and Pb(II) at each Ag-NPs-SPCNFE in the selected concentration range (data not shown), being for all cases an E_d_ of −1.40 V applied with stirring during a t_d_ of 120 s and followed by a t_r_ of 5 s.

**Figure 6:**
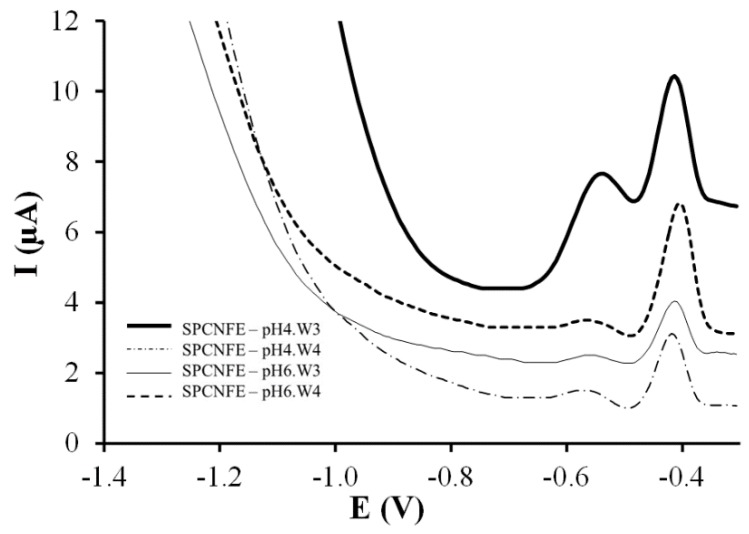
Stripping voltammetric measurements of the SPCNFE modified with Ag-NPs obtained from different synthesis pH conditions and washing suspensions for the simultaneous detection of Cd(II) and Pb(II) at 77 µg L^−1^ and pH 4.5.

The comparison of the analytical performance of the SPCNFE modified with Ag-nanoparticles obtained from different synthesis pH conditions and after two or three additional rinsing suspensions (W3 and W4) is shown in Figure 6. In order to study the effect of the NP size on the electrochemical behavior and to avoid the presence of other compounds in solution, Ag-NPs obtained at pH 4 and 6 were chosen to prepare SPCNFE. The aim of the modified Ag-NPs-SPCNFE is the simultaneous determination of a solution containing Pb(II) and Cd(II). Well-defined peaks can be observed for both Pb(II) and Cd(II) ions at −0.42 and −0.55 V, respectively, with all electrodes. However, more intense peaks are obtained using the SPCNFE modified with Ag-NPs prepared at pH 4, supernatant W3 (SPCNFE—pH4.W3), particularly in the case of Cd(II).

**Figure 7:**
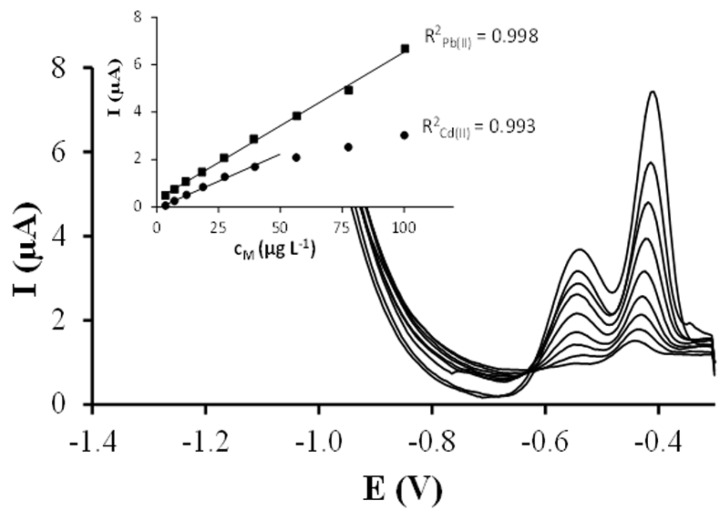
DPASV measurements and calibration curves (insets) obtained for the simultaneous calibration of Pb(II) and Cd(II) in acetate buffer pH 4.5 using an Ag-NPs modified -SPCNFE at an E_d_ of −1.4 V and a t_d_ of 120 s.

Simultaneous calibration of Pb(II) and Cd(II) ions by DPASV were carried out on each Ag-NPs-SPCNFE. For this purpose, nine increasing concentrations of Pb(II) and Cd(II) ranging from 1.1 to 100.4 and from 1.1 to 100.1 µg L^−1^, respectively, were used as calibration samples. Figure 7 shows, as an example, the evolution of the DPASV signals of both metal ions using SPCNFE modified with silver nanoparticles obtained from SPCNFE—pH4.W3 when their concentrations increase (the other three Ag-NPs-SPCNFE present equivalent behaviors). The obtained calibration data on each Ag-NPs-SPCNFE are summarized in Table 2. The limit of detection (LOD) was calculated as three times the standard deviation of the intercept over the slope of the calibration curve of the target ions. The limit of quantification (LOQ) was evaluated by considering 10 times the previous ratio. The lowest value of the linear concentration range was established from the corresponding LOQ. As shown in Table 2, linear calibration curves were obtained for Pb(II) and Cd(II) up to a maximum concentration level of 100.4 and 27.5–39.7 μg L^−1^, respectively, depending on the considered Ag-NPs-SPCNFE. Regarding sensitivities (nA µg^−1^ L), obtained from the slope of the calibration curves, they varied from 21.2 to 62 for Pb(II) and from 5.1 to 46 for Cd(II) depending on the Ag-NPs-SPCNFE (Table 2). The LOD of the determination of the two metals in the four considered Ag-NPs-SPCNFEs ranged from 2.7 to 4.9 for Pb(II) and from 2.8 to 8.1 for Cd(II) according to the considered Ag-NPs-SPCNFE, whereas the LOQ varied from 8.9 to 16.2 for Pb(II) and from 9.5 to 26.9 for Cd(II) (as seen in Table 2). It should be mentioned that no previous LOD and LOQ data for SPCNFEs modified with Ag-NPs from natural sources are available in the literature. Nevertheless, as compared to previous results reported using other electrodes, e.g., synthetic Ag-NPs-based electrodes [16,32], bismuth film electrodes [33], antimony film electrodes [34], chemically modified electrodes [35,36,37,38], the LOD and LOQ achieved in this work for Cd(II) and Pb(II) are similar or even lower depending on the considered modified electrode.

Therefore, the reported calibration data suggests that all the considered Ag-NPs-SPCNFEs could be suitable and a valuable option for more conventional electrodes for the simultaneous determination of Pb(II) and Cd(II) at trace levels in natural samples. However, the best results (higher sensitivities, lower LODs and wider linear ranges) were obtained with SPCNFE-pH4.W3.

## 4. Conclusions

An environmentally friendly preparation of Ag-NPs by using non-toxic reducing chemicals has been developed. Thus, total polyphenols and reducing sugars present in plant extracts from grape stalk wastes were used as reducing agents and stabilizers in the nanoparticles production. The extract and the Ag-NPs generation have been studied and optimized considering several parameters and have been characterized in detail by different spectroscopic and electron microscopy techniques. Finally, these Ag-NPs were drop-casted on the SPCNFE and the resulting sensors were analytically characterized to proof their suitability for sensing purposes. These modified Ag-NPs-SPCNFE were tested for the simultaneous stripping voltammetric determinations of Pb(II) and Cd(II), providing appropriate reproducibility, sensitivity, linear range and LODs at µg L^−1^ range; more precisely, LOD_Pb(II)_ 2.7 µg L^−1^ and LOD_Cd(II)_ 2.8 µg L^−1^, evidencing the enhancement of the SPCNFE electrodes by adding the above mentioned Ag-NPs.

## Figures and Tables

**Table 1:** Diameter measurements of the Ag-NPs prepared at different pH obtained from TEM micrographs.

Sample Preparation pH	Diameter (nm)
2	55.4 ± 0.3
4	27.7 ± 0.6
6	54.3 ± 0.1
8	9.0 ± 0.2

**Table 2:** Calibration data for the simultaneous determination of Pb(II) and Cd(II) at each Ag-NPs-SPCNFE at E_d_ of −1.4 V, t_d_ of 120 s and pH 4.5. Standard deviations are denoted by parenthesis.

Sample	Analytical Parameter	DPASV Calibration	
		Pb(II)	Cd(II)
SPCNFE—pH4.W3	Sensitivity (nA µg^−1^ L)	62 (1)	46 (2)
	R^2^	0.998	0.993
	Linear range ^a^ (µg L^−1^)	8.9–100.4	9.5–39.7
	LOD (µg L^−1^)	2.7	2.8
SPCNFE—pH4.W4	Sensitivity (nA µg^−1^ L)	26.0 (0.7)	8.5 (0.5)
	R^2^	0.996	0.993
	Linear range ^a^ (µg L^−1^)	13.3–100.4	16.0–39.7
	LOD (µg L^−1^)	4.0	4.8
SPCNFE—pH6.W3	Sensitivity (nA µg^−1^ L)	21.2 (0.5)	5.1 (0.5)
	R^2^	0.996	0.980
	Linear range ^a^ (µg L^−1^)	12.3–100.4	26.9–39.7
	LOD (µg L^−1^)	3.7	8.1
SPCNFE—pH6.W4	Sensitivity (nA µg^−1^ L)	43 (1)	13.8 (0.8)
	R^2^	0.993	0.989
	Linear range ^a^ (µg L^−1^)	16.2–100.4	10.0–27.5
	LOD (µg L^−1^)	4.9	3.0

^a^ The lowest value of the linear range was considered from the LOQ.
